# Mediation of a GDSL Esterase/Lipase in Carotenoid Esterification in Tritordeum Suggests a Common Mechanism of Carotenoid Esterification in Triticeae Species

**DOI:** 10.3389/fpls.2020.592515

**Published:** 2020-12-17

**Authors:** María Dolores Requena-Ramírez, Sergio G. Atienza, Dámaso Hornero-Méndez, Cristina Rodríguez-Suárez

**Affiliations:** ^1^Instituto de Agricultura Sostenible ‐ Consejo Superior de Investigaciones Científicas, Córdoba, Spain; ^2^Department of Food Phytochemistry, Instituto de la Grasa ‐ Consejo Superior de Investigaciones Científicas, Campus Universidad Pablo de Olavide, Seville, Spain

**Keywords:** carotenoids, *H. chilense*, lutein esters, tritordeum, XAT, xanthophyll esterification

## Abstract

Carotenoids are essential in human diet, so that the development of programs toward carotenoid enhancement has been promoted in several crops. The cereal tritordeum, the amphiploid derived from the cross between *Hordeum chilense* Roem. et Schulz. and durum wheat has a remarkable carotenoid content in the endosperm. Besides, a high proportion of these carotenoids are esterified with fatty acids. The identification of the gene(s) responsible for xanthophyll esterification would be useful for breeding as esterified carotenoids show an increased ability to accumulate within plant cells and have a higher stability during post-harvest storage. In this work, we analyzed five genes identified as candidates for coding the xanthophyll acyltransferase (XAT) enzyme responsible for lutein esterification in *H. chilense* genome. All these genes were expressed during grain development in tritordeum, but only HORCH7HG021460 was highly upregulated. Sequence analysis of HORCH7HG021460 revealed a G-to-T transversion, causing a Glycine to Cysteine substitution in the protein of H290 (the only accession not producing quantifiable amounts of lutein esters, hereinafter referred as zero-ester) of *H. chilense* compared to the esterifying genotypes. An allele-specific marker was designed for the SNP detection in the *H. chilense* diversity panel. From the 93 accessions, only H290 showed the T allele and the zero-ester phenotype. Furthermore, HORCH7HG021460 is the orthologue of XAT-7D, which encodes a XAT enzyme responsible for carotenoid esterification in wheat. Thus, HORCH7HG021460 (XAT-7Hch) is a strong candidate for lutein esterification in *H. chilense* and tritordeum, suggesting a common mechanism of carotenoid esterification in Triticeae species. The transference of XAT-7Hch to wheat may be useful for the enhancement of lutein esters in biofortification programs.

## Introduction

Carotenoids pigments are responsible for many of the yellow, orange, and bright red colors of fruits, vegetables, and flowers. They have essential roles in light-harvesting, protection against excess of light energy and oxidative damage (Britton, 2008). Humans are not able to synthesize carotenoids and thus their supply relies on diary intake. The consumption of carotenoid-rich foods has been associated with a lower risk of developing some diseases, including several types of cancer (Nishino et al., 2009). Vitamin A deficiency is a serious concern in developing countries since it leads to increased childhood mortality, growth retardation, and blindness (WHO, 2009). All carotenoids with at least one unsubstituted β-end ring have provitamin A activity, and consequently, β-carotene is the main vitamin A precursor (Giuliano et al., 2008). Lutein has also been suggested to play an important role during neural system development in infant brains (Johnson, 2014). In addition, lutein and zeaxanthin are particularly concentrated in the eye macula, i.e., the central part of the retina (Johnson, 2014), and their consumption alleviates age macular degeneration (AMD; Krinsky et al., 2003).

In wheat and related cereals, lutein is the main carotenoid in grains (Rodríguez-Suárez et al., 2010; Ficco et al., 2014). Tritordeum (×*Tritordeum martini* A. Pujadas; Pujadas-Salvá, 2016) is the amphiploid derived from the cross between *Hordeum chilense* Roem. et Schultz. and durum wheat, and it has an unusually high carotenoid content (Martín et al., 1999). On average, tritordeums have over five times more carotenoids in seeds than their corresponding durum wheat parents (Atienza et al., 2007b). Thus, this species is suitable for the development of biofortification programs as in the case of maize (Menkir et al., 2018; Zhu et al., 2018) and rice (Paine et al., 2005). Tritordeum is being commercialized by Agrasys S.L.^1^ At present, tritordeum products are available in many European countries and Australia.^2^

The carotenoid biosynthesis pathway in plants is well-known (Rodriguez-Concepcion et al., 2018). The first step is regulated by phytoene synthase (PSY). In grasses, there are three *Psy* genes, *Psy1*, *Psy2*, and *Psy3* (Li et al., 2009), but only *Psy1* contributes to endosperm carotenoid accumulation (Li et al., 2009). The same happens in tritordeum, with *Psy1_Hch* being the main responsible for the endosperm carotenoid content (Atienza et al., 2007a; Rodríguez-Suárez et al., 2014; Mattera et al., 2015).

In addition to carotenoid content, the bioavailability of these pigments and its retention through the food chain are of the outmost importance (Fernández-García et al., 2012). Lutein esters have been found to be more stable than free lutein at 60°C (Ahmad et al., 2013). Indeed, collective evidence show that esterification increases carotenoids stability and accumulation (Mellado-Ortega and Hornero-Méndez, 2015b; Meléndez-Martínez et al., 2019); thus, improving carotenoid esterification in cereal endosperm would be useful for biofortification programs.

Tritordeum contains a high proportion of lutein esters in the endosperm, while durum wheat produces low amounts (Atienza et al., 2007b) or no lutein esters at all (Ziegler et al., 2015). Accordingly, this high proportion of lutein esters found in tritordeum was suggested to be caused by genes from the *H. chilense* genome (Hch). This hypothesis was confirmed by analyzing *H. chilense* seeds (Mellado-Ortega and Hornero-Méndez, 2015a) and *H. chilense*-common wheat genetic stocks (Mattera et al., 2015). Furthermore, these results showed the existence of genes for lutein esterification in both chromosomes 7Hch from *H. chilense* and 7D from common wheat (Mattera et al., 2015). The presence of genes for lutein esterification in the D genome from common wheat is consistent with the diversity observed for carotenoid esterification in this species (Ziegler et al., 2015; Paznocht et al., 2019).

Using a 7D chromosome deletion set in common wheat, along with the reference genome (cv. “Chinese Spring”) and the Wheat Expression Browser (Ramírez-González et al., 2018), we identified the target region harboring the gene(s) for lutein esterification in chromosome 7D (Ávila et al., 2019b). This allowed for the selection of a set of candidate genes with xanthophyll acyltransferase (XAT) function, which could be responsible for the *Lute* locus associated to lutein esterification in the cross Haruhikari//Sunco/Indis.82 (Ahmad et al., 2015). Due to the high degree of genetic collinearity that *H. chilense* shows with Triticeae species (Ávila et al., 2019a), the set of candidate genes identified in chromosome 7D is also valid for chromosome 7Hch, since orthologs of these genes are likely present in chromosome 7Hch. Indeed, genetic studies have shown that orthologous genes for traits of interest are in equivalent positions in *H. chilense* compared to wheat and/or barley, for instance *Psy1* (Atienza et al., 2007a), APETALA2-like (Gil-Humanes et al., 2009), carotenoid biosynthesis pathway (Rodríguez-Suárez and Atienza, 2012), *Ppo* (Rodríguez-Suárez and Atienza, 2014), *Wx* (Alvarez et al., 2019), and *Rfm1* (Rodríguez-Suárez et al., 2020).

The objectives of this work were to identify the orthologue genes of *H. chilense* corresponding to a set of candidate genes with putative XAT function in chromosome 7D, to investigate their expression profile during grain development in tritordeum, and to identify the gene responsible for lutein esterification using a diversity panel of *H. chilense*.

## Materials and Methods

### Candidate Gene Identification and Amplification in *H****ordeum*** *chilense*

*Hordeum chilense* (H7 and H16), wheat (*Triticum aestivum* “Chinese Spring”), and barley (*Hordeum vulgare* “Betzes”) seeds were sown in pots with a mixture 1:4.5 (v:v; sand:premium pflanzerde substrate). Osmocote Exact Mini (1.2 g/L) Everris International B.V. was added. Two-week-old leaves were used for DNA isolation. Genomic DNA was extracted using the CTAB method according to Murray and Thompson (1980) with slight modifications.

A set of five candidate genes located in the homeologous group 7 of the Triticeae species were selected (Table 1). This set of genes was selected from a previous genetic study (Ávila et al., 2019b). The annotation of these genes is related to the esterification process, and all of them are expressed during grain development in common wheat as determined using the Wheat Expression Browser powered by expVIP (Borrill et al., 2016; Ramírez-González et al., 2018). The sequences of these genes in wheat were retrieved from Ensembl Plants website and used to identify the orthologous genes in barley (Howe et al., 2019; Additional File 1, Supplementary Table S1).^3^

**Table 1:** Candidate genes for lutein esterification.

Gene	Function	Location in 7D (Mb)
TraesCS7D02G011600	Dihydrolipoyllysine-residue acetyltransferase component of PDC	5,164,729–5,170,165
TraesCS7D02G037200	GDSL_esterase/lipase	18,880,746–18,884,635
TraesCS7D02G093200	GDSL_esterase/lipase	55,428,466–55,430,682
TraesCS7D02G094000	GDSL_esterase/lipase	56,402,415–56,404,256
TraesCS7D02G108900	GDSL_esterase/lipase	65,599,420–65,601,414

PDC, Pyruvate dehydrogenase complex.

To obtain *H. chilense* genomic sequences, the wheat (cv. Chinese Spring) and barley (cv. Morex) sequences available for each gene at Ensembl Plants were aligned using the multiple sequence alignment tool ClustalW (Madeira et al., 2019). Primers were designed in the conserved regions to ensure amplification in *H. chilense* templates (Additional File 1, Supplementary Table S2). The NCBI Primer-Blast tool was used for primer design (Ye et al., 2012). PCR amplifications were carried out with MyTaq™ DNA polymerase (Bioline, London, UK) following the manufacturer’s instructions, using H7 and H16 *H. chilense* accessions, along with barley (cv. Betzes) and wheat (cv. Chinese Spring) samples as controls. Amplification products were resolved in agarose gels and visualized with Safeview™ Nucleic Acid Stain (NBS Biologicals, Ltd., Cambridgeshire, England). The PCR fragments obtained were directly sequenced at STAB VIDA (Portugal).^4^

### Quantitative Real-Time PCR During Grain Development

Tritordeum samples used for quantitative real-time PCR (qPCR) experiments were obtained in a previous work (Rodríguez-Suárez et al., 2014). Briefly, five hexaploid tritordeums [HT240, HT335, HT609, HT621 (Ballesteros et al., 2005), and HT630] were grown at field conditions following a completely randomized design with two replicates. Samples for qPCR analyses were obtained from developing grains of tritordeums. Spikes were marked at anthesis and developing grains were collected with forceps at 8 (Zadoks 71), 14 (Zadoks 73), 18 (Zakoks 77), and 25 (Zakoks 83) days post-anthesis (dpa; Zadoks et al., 1974) and immediately frozen in liquid nitrogen at −80°C. From each block, two independent biological replicates were collected (total of four biological replicates, two samples per block and two blocks in the field trial). From each biological replicate, four grains were randomly selected for RNA extraction. Thus, eighty samples were analyzed (five genotypes × four developmental stages × four biological samples).

Total RNA was isolated from whole grains using the TRIzol® Reagent (Invitrogen, Carlsbad, CA) according to manufacturer’s instructions with slight modifications, and cDNA was obtained as described previously (Rodríguez-Suárez et al., 2011).

Once *H. chilense* partial sequences were obtained for the five genes, RT-qPCR primers were designed to specifically amplify *H. chilense* gene copy in tritordeum. For this purpose, sequence sets for each gene were aligned and primers were designed as mentioned above considering polymorphic regions. Primer pair amplification specificity was checked by the standard PCR.

Real-time qPCR reactions for target genes were carried out using four biological replicates with two technical duplicates each from cDNA samples, which is consisted in a 1:4 dilution of each sample. Amplicon sizes ranged from 99 to 155 bp. RT-qPCR reactions were carried out using the SYBR® Green on an Applied Biosystems™ 7500 Real-Time PCR System (Applied Biosystems, Foster City, CA, USA). The PCR conditions were 95°C for 10 min followed by 40 cycles at 95°C for 15 s and 60°C for 1 min. qPCR reactions were performed using 5 μl of diluted cDNA (10 ng/μl), 5 μl of iTaqTM Universal SYBR® Green Supermix (Bio-Rad, Hercules, California), and a primer pair concentration of 0.22 μM each. No template controls were included in the reactions.

Homeolog-specific amplification was confirmed by the presence of a single peak in the qPCR dissociation curve analysis. PCR efficiency of each primer pair was determined by the LinRegPCR quantitative PCR data analysis program (version 11.0; Ruijter et al., 2009) using raw normalized fluorescence as input data. Expression of target genes for each sample (N_0_) was determined using the equation N_0_ = 0.2/E^Cq^, where E is the PCR efficiency for each primer and Cq is the number of cycles needed to reach 0.2 arbitrary units of fluorescence.

Geometric mean of reference genes ADP-RF(m), RLI(a), and CDC(a) was used for normalization (Giménez et al., 2011). Expression stability of these genes and normalization factors for each sample were assessed using GeNorm (Vandesompele et al., 2002).

### SNP Detection in HORCH7HG021460 Gene

Three *H. chilense* genotypes were used to obtain the complete sequence of this gene, H7, H16, and H290, the only *H. chilense* accession not producing quantifiable amounts of lutein esters (hereinafter referred as zero-ester genotype; Ávila et al., 2019a). For the complete DNA genomic sequencing of HORCH7HG021460, three overlapping fragments were amplified, sequenced directly from PCR products and assembled (Additional File 2, Supplementary Figure S1).

SignalP 4.0 software was used for signal peptide and cellular location prediction of the expected proteins (Petersen et al., 2011). Search for conserved domain analysis was carried out by using the Conserved Domain Database (Lu et al., 2019).

A SNP was identified between H290 (zero-esters) and H7, H16. Primer 1 web service was used for primer design, following a Tetra-Primer ARMS (amplification refractory mutation system) strategy (Collins and Ke, 2012) for SNP detection in HORCH7HG021460: Fw_out GCTTACTGCTCCTGGCCATCTTCCTC; Rv_out TTGAACCCAATCTTTTCAGCTGCAACAA; Fw_inn_H290 CATGTCGCCACAGGGAGGTTCTGCAGCT; Rv_inn_wild AACCTATGAAATCGATAAGCAGCTTACC.

A diversity panel of 93 accessions of *H. chilense*, previously characterized for carotenoid content and profile (Ávila et al., 2019a), was used for the validation of the SNP detected in this work. The carotenoid content and profile of these accessions are shown in Additional File 3. The origin of these accessions has been previously reported (Vaz Patto et al., 2001).

## Results

### Candidate Genes Identification in *H****ordeum*** *chilense*

To identify *H. chilense* orthologs of the candidate genes for lutein esterification in the D genome (Table 1), wheat homeologues, along with *H. vulgare* orthologs, were identified using the wheat reference genome (Additional File 1, Supplementary Table S1). Gene sequences were aligned and used as a template for primer design considering the conserved regions. Amplification products obtained in *H. chilense* accessions H7 and H16 were directly sequenced. The identity of the *H. chilense* sequences obtained was confirmed by BLASTn, showing high homologies with the corresponding orthologue genes (Additional File 1, Supplementary Table S3). The five genes were then named based on *H. vulgare* gene’s ID: HORCH7HG001330, HORCH7HG007590, HORCH7HG021310, HORCH7HG021460, and HORCH7HG024220. Partial sequences of these genes were used along with those from barley and wheat to design gene-specific primers for RT-qPCR (Additional File 1, Supplementary Table S2). These primers only amplified *H. chilense* copies as confirmed by conventional PCR.

### Transcript Profiling of *H****ordeum*** *chilense* Candidate Genes for Lutein Esterification in Tritordeum

**Figure 1:**
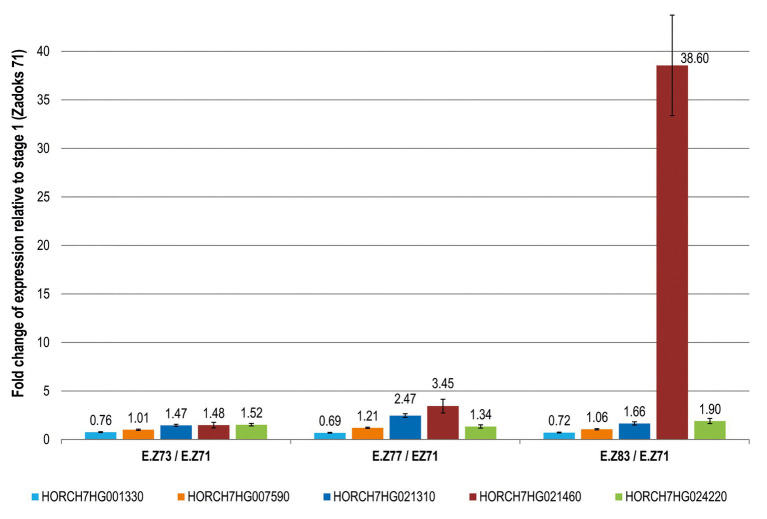
Fold change expression of candidate genes during grain development in tritordeum. For each gene (represented by a different color), the fold change of expression at each developmental stage was calculated relative to stage 1 (Zadoks 71). Ratios above 1 indicate upregulation of the gene compared to the first stage (Zadoks 71). The gene HORCH7HG001330 has ratios below 1, which indicates a down regulation of this gene compared to the first stage (Zadoks 71). Bars were calculated as the mean of five different tritordeum genotypes ± SE.

Tritordeum samples from four grain developing stages (St) were used for transcript profiling: St1 [Zadoks 71, 8 days post-anthesis(dpa)]; St2 (Zadoks 73, 14 dpa); St3 (Zadoks 77, 18 dpa) and St4 (Zadoks 83, 25 dpa; Zadoks et al., 1974). These developmental stages correspond to the caryopsis water ripe (Z71), early milk (Z73), late milk (Z77) and early dough (Z83; Zadoks et al., 1974). Several primer pairs were designed for each candidate gene, selecting those with specific amplification (Additional File 1, Supplementary Table S2). For each gene, the change in expression was calculated relative to the expression at St1 (Figure 1) considering the mean values and standard errors calculated with all tritordeum samples (five genotypes × two biological replicates × two field plots). The five *H. chilense* genes were expressed during grain development but they showed great differences in their expression patterns (Figure 1). Indeed, HORCH7HG021460 increased remarkably its relative abundance during grain development with a 38.6-fold up-regulation in St4 relative to St1 (Figure 1). All the other genes were in the range 0.7–2.0 relative to St1 with the exception of HORCH7HG021310 which showed a 2.5 fold increase in St3.

### Sequence Analysis in a *H****ordeum*** *chilense* Accession Unable to Synthesize Lutein Esters

**Figure 2:**
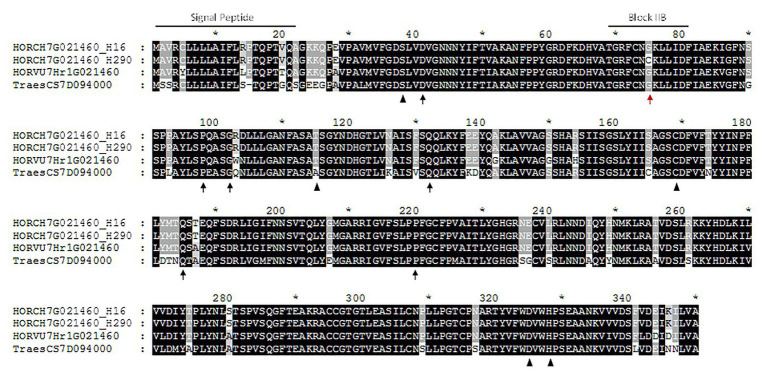
Alignment of XAT proteins. Protein alignment of HORCH7HG021460 of *Hordeum chilense* accessions H16 (identical to H7) and H290, HORVU7Hr1G021460 of *Hordeum vulgare* and TraesCS7D094000 of bread wheat. The predicted active sites are shown with black triangles. Black arrows show the mutations described in Gladius TILLING population (Watkins et al., 2019). The mutation detected in the zero-ester genotype H290 is pointed with a red arrow, within the conserved consensus sequence of block IIB (Akoh et al., 2004). Signal peptide in positions 1–22 is also highlighted in HORCH7HG021460 proteins.

This high increase in HORCH7HG021460 expression at the end of the grain development may indicate a putative role in lutein esterification. Recently we identified the genotype H290, a *H. chilense* accession without quantifiable amounts of lutein esters (zero-ester genotype; Ávila et al., 2019a). We obtained the complete gene sequence of HORCH7HG021460 in H290 and compared it to two *H. chilense* esterifying genotypes H7 and H16. For that purpose, a set of three primers pairs were designed (Additional File 1, Supplementary Table S2) to amplify overlapping fragments and obtain the gene sequence in the selected genotypes. The intron/exon structure was determined based on that of *H. vulgare* gene HORVU7Hr1G021460. The *H. chilense* gene was predicted to have a five exon structure, with ORFs of 1,462 bp in H290 and 1,460 bp in H7 and H16. A 22 amino acid-length signal peptide was found in the sequences of H7, H16, and H290, predicting an extracellular protein location. HORCH7HG021460 encodes for a 352 amino acid-length GDSL protein (characterized by the conserved GDSL motif), where the active sites at positions 38 (S), 115 (T), 169 (C), 327 (D), and 330 (H) have been identified (Figure 2). After comparing the coding sequences, a single SNP was found at the end of exon 1: a G in H7 and H16 and a T in H290 (Supplementary Figure S1; Additional File 2). In the predicted proteins, this transversion causes a Glycine (Gly) to Cysteine (Cys) amino acid change at position 75 in zero-ester genotype (Figure 2).

### Validation of the SNP for Zero-Ester in the *H****ordeum*** *chilense* Diversity Panel

A tetra-Primer ARMS strategy was optimized to detect the G/T SNP in gene HORCH7HG021460. The outer primer pair amplified a common fragment of 344 bp in all genotypes. A 163 bp fragment amplified in the allele present in H290 whereas a 237 bp fragment was obtained in H7 and H16 genotypes. The SNP was scored in the *H. chilense* diversity panel composed by 93 accessions, previously characterized for their carotenoid and esterification profile (Ávila et al., 2019a). All lines producing esters (92) showed the 237 bp allele, and only H290 showed the 163 bp allele and the zero-ester phenotype.

## Discussion

The genes studied in this work were initially identified as candidates to explain the underlying variation for lutein esterification in chromosome 7D of common wheat (Ávila et al., 2019b). They were selected considering their annotated function, their position in the “Chinese Spring” genome, and their expression profile retrieved from the Wheat Expression Browser. *Hordeum chilense* shows a good degree of collinearity with *H. vulgare* (Ávila et al., 2019a) and, thus, with all the members of the Triticeae tribe (Mayer et al., 2011). Indeed, the position of *H. chilense* genes identified using candidate gene approaches in previous works is in agreement with their position in other Triticeae species (Atienza et al., 2007a; Gil-Humanes et al., 2009; Rodríguez-Suárez and Atienza, 2012; Alvarez et al., 2019; Rodríguez-Suárez et al., 2020). Thus, these candidate genes in chromosome 7D were a good starting point for identifying the gene involved in lutein esterification in Hch genome.

One of the hypotheses to be tested was if the orthologue genes from *H. chilense* were expressed during grain development, as it happens with the candidate genes from D genome in wheat. In fact, all the candidate genes tested were expressed during grain development with different expression patterns (Figure 1), among which HORCH7HG021460 showed a remarkable upregulation. Interestingly, its wheat orthologue TRAESCS7D02g094000 (*XAT-7D*) has been recently identified as the gene coding for the XAT enzyme responsible for lutein esterification in common wheat (Watkins et al., 2019). As observed for *XAT-7D*, HORCH7HG021460 encodes for a GDSL protein, predicted to have an extracellular (apoplastic) location based on the conserved domain and signal peptide analyses.

To clear up whether HORCH7HG021460 may be associated with variation for lutein esterification in *H. chilense*, we studied the accession H290, the only zero-ester member within the species diversity panel (Ávila et al., 2019a). Genomic sequence of HORCH7HG021460 in H290 was compared with those of the two selected esterifying accessions, H7 and H16. This analysis revealed a mutation leading to an amino acid change from Gly to Cys at position 75 in the H290 protein (Figure 2). This position takes part of the conserved domain for block IIB (TGRF-NG----D--) defined for GDSL proteins by Akoh et al. (2004). Although this amino acid position is not identified as an active site, it is highly conserved among distant species (Additional File 4, Supplementary Figure S2), which may suggest its importance in the carotenoid esterification protein activity. Watkins et al. (2019) analyzed a TILLING population derived from EMS-mutagenized wheat cultivar Glaudius and found six independent mutations leading to the absence of lutein esters. None of these mutations correspond to the active sites defined for GDSL proteins (Figure 2), and they are different from that found in H290. Thus, not only mutations in the active sites may lead to non-functional GDSL proteins but the mutation found in accession H290 may also explain the zero-ester phenotype. Additionally, all accessions from the diversity panel harbor the “G” allele (237 bp fragment) of HORCH7HG021460 and are able to produce esters, and only H290 shows both the zero-ester phenotype and the “T” allele (163 bp fragment). Thus, we propose HORCH7HG021460 (*XAT-7Hch*) as the gene responsible for lutein esterification in *H. chilense* and tritordeum. Although a functional analysis would be necessary for further confirmation, the findings in the diversity panel and the functional validation of the wheat ortholog *XAT-7D* are strong evidences supporting the role of *XAT-7Hch* in the esterification of lutein in tritordeum.

Here, we provide evidence that the same XAT mechanism may be acting in both D and Hch genomes. This is relevant for Triticeae species since it points out to a general mechanism of carotenoid esterification useful for cereals biofortification. Indeed, the simultaneous presence of chromosomes 7D and 7Hch (carrying *XAT-7D* and *XAT-7Hch*, respectively) in *H. chilense*-wheat chromosome substitution lines results in higher amounts of lutein esters (Mattera et al., 2015). This may indicate an additive effect of *XAT-7D* and *XAT-7Hch*, which is in agreement with previous results, suggesting that the amount of lutein esters may be limited by the existence of specific XAT enzymes and not by substrate availability (Mellado-Ortega and Hornero-Méndez, 2018).

Our collective data suggest some similarities and some differences between the XAT enzymes in bread wheat and *H. chilense*. Watkins et al. (2019) predicted XAT to be localized to the apoplast, while carotenoids are produced in plastids (Shumskaya et al., 2012). As a consequence, these authors hypothesized that this subcellular partitioning of substrates and enzymes would constitute a mechanism of regulation of lutein esterification in wheat grain throughout time. The signal peptide of *XAT-7Hch* also predicts an apoplastic location, which is in agreement with our observations. In a previous work, we investigated the carotenoid profile from 20 to 40 dpa in developing seeds of tritordeum and in three common wheat-*H. chilense* chromosome substitution lines carrying chromosome 7Hch (Mattera et al., 2020). Despite the high amounts of carotenoids, no lutein esters were detected until 36 dpa (Mattera et al., 2020), when desiccation had been initiated and endosperm cells would be under their programmed cell death, resulting in the loss of membrane integrity (Brown and Lemmon, 2007). From this point onward, carotenoids may be accessed by XAT-7Hch, resulting in the production of carotenoid esters.

As it has been found for most fruits, the formation of xanthophyll esters during fruit ripening is characterized for a lack of correlation between the fatty acid profile of the total lipid pool and the major fatty acids involved in the esterification, suggesting that the responsible enzyme is rather specific toward the acyl group (Hornero-Méndez, 2019). Regarding XAT-7D specificity, Watkins et al. (2019) reported a good correlation between the fatty acid abundance in the endosperm and the fatty acids esterified to lutein in endosperm in wheat, which would indicate that XAT from bread wheat has low discrimination for the fatty acids. In the case of tritordeum, palmitic acid (16:0) and linoleic acid (18:2) are the fatty acids involved in the esterification of lutein, with a general preference for palmitic acid (16:0) being observed (Mellado-Ortega and Hornero-Mendez, 2012; Mellado-Ortega et al., 2015; Mattera et al., 2017) despite linoleic acid (18:2) being the most abundant in tritordeum endosperm (Mellado-Ortega and Hornero-Méndez, 2018) as it happens in bread wheat (Ziegler et al., 2015) and other Triticeae species. This suggests a higher specificity of XAT-7Hch compared to XAT-7D from bread wheat. Indeed, in a wide survey for carotenoid esterification patterns in *H. chilense*, the majority of accessions showed preference toward palmitic acid or similar preference toward palmitic and linoleic acids, while only three accessions used linoleic as the preferred fatty acid for lutein esterification (Ávila et al., 2019a).

In conclusion, a set of *H. chilense* genes putatively involved in xanthophylls esterification were successfully identified, and its expression during grain development in tritordeum was confirmed. Among them, HORCH7HG021460, referred as *XAT-7Hch*, seems to be responsible for the esterification of lutein in *H. chilense*. Thus, lutein esterification in *H. chilense* and tritordeum would be regulated by the same enzymatic system than in bread wheat suggesting the existence of a common mechanism for lutein esterification in the Triticeae. The transference of *XAT-7Hch* to durum or bread wheat may allow the improvement of their nutritional value by increasing the carotenoid accumulation and improving their stability and retention during post-harvest storage of grains. The SNP marker developed in this work is useful for a marker-assisted selection (MAS) strategy with this purpose. Further studies will be needed for determining the specificity of XAT-7Hch and its potential application in tritordeum breeding programs.

## Data Availability Statement

The datasets presented in this study can be found in online repositories. The names of the repository/repositories and accession number(s) can be found below: GenBank accessions MT880902, MT880903, and MT880904.

## Author Contributions

SA and DH-M conceived the idea. CR-S and SA developed the field experiment and collected the field samples. MR-R and CR-S developed the molecular studies. All authors contributed to the analysis and interpretation of the results, the redaction of the manuscript draft and approved the final version.

### Conflict of Interest

The authors declare that the research was conducted in the absence of any commercial or financial relationships that could be construed as a potential conflict of interest.

## Footnotes

^1^https://www.agrasys.es/en/

^2^https://www.tritordeum.com/ww/?lang=en, last accessed 11/11/2020.

^3^https://plants.ensembl.org/

^4^www.stabvida.com
