# Supplementary material for: Mediation of a GDSL Esterase/Lipase in Carotenoid Esterification in Tritordeum Suggests a Common Mechanism of Carotenoid Esterification in Triticeae Species
Source: Front Plant Sci. 2020 Dec 17;11:592515. doi: 10.3389/fpls.2020.592515 (PMC7971304; doi:10.3389/fpls.2020.592515)

Additional file 2. Requena-Ramírez et al.

Figure S1. Nucleotide alignment of the genomic (gDNA) and coding (CDS) sequences of the *H. vulgare* gene HORVU7Hr1G021460 (Hv), and the genomic sequences of the allelic variants of gene HORCH7HG21460 in H7, H16 and H290 *H.chilense* accessions. The only SNP detected in the coding region among *H. chilense* alleles is highlighted. *H. chilense* sequence data have been submitted to NCBI (H7 →MT880902; H16 → MT880903; H290 →MT880904).

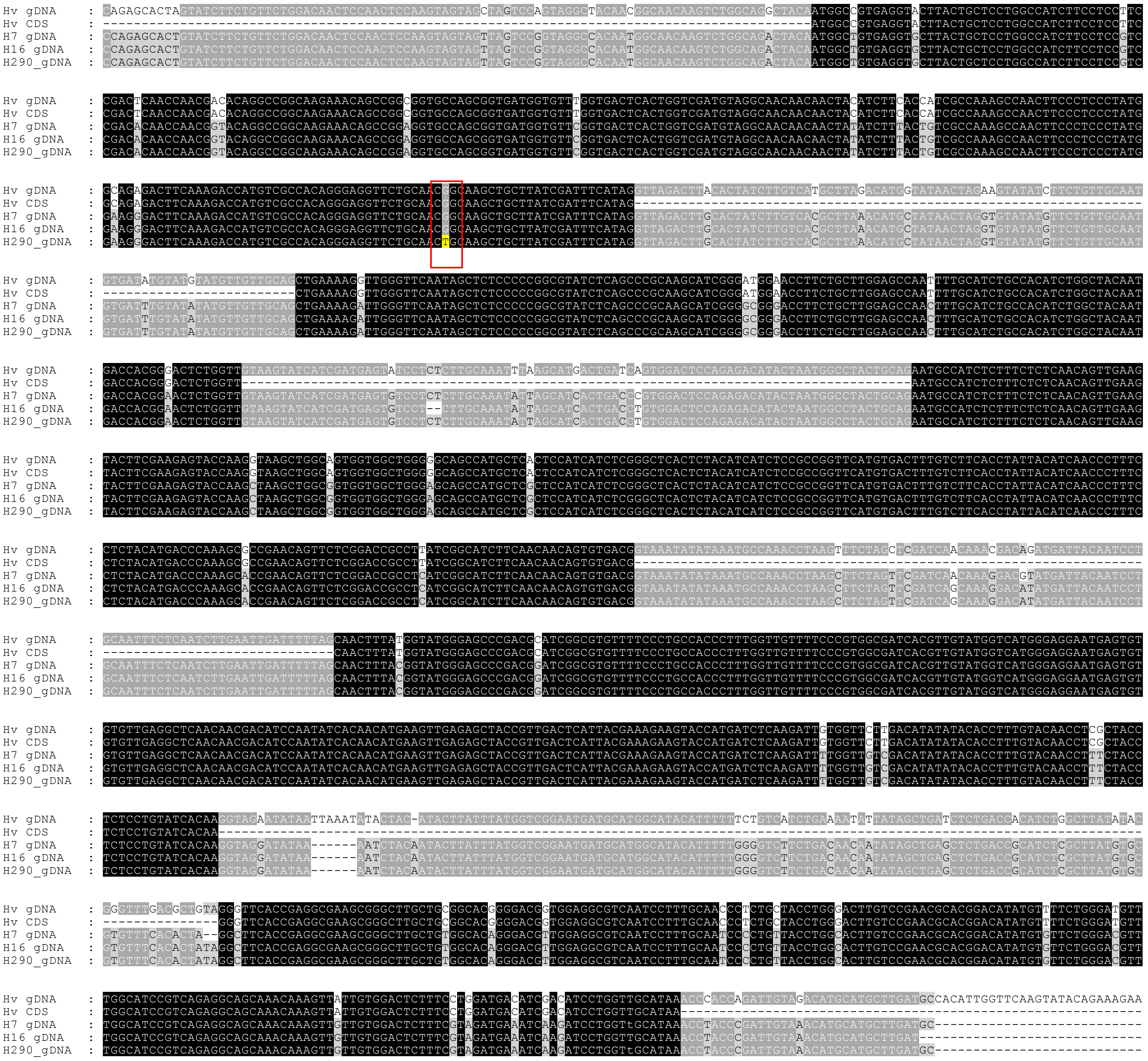

Supplement: Supplementary file 1 [file Data_Sheet_1.pdf]
