# Supplementary material for: Mediation of a GDSL Esterase/Lipase in Carotenoid Esterification in Tritordeum Suggests a Common Mechanism of Carotenoid Esterification in Triticeae Species
Source: Front Plant Sci. 2020 Dec 17;11:592515. doi: 10.3389/fpls.2020.592515 (PMC7971304; doi:10.3389/fpls.2020.592515)
Supplement: Supplementary file 2 [file Data_Sheet_2.pdf]

Requena-Ramírez et al. Additional file 3. Geographic localization and carotenoid composition in 93 accessions of *H. chilense (data from Ávila et al. 2019. https://link.springer.com/article/10.1007%2Fs10681-019-2369-6)*

GENOTYPE	Latitiud	Longitud	Lutein monoesters	Lutein diesters	Total Carotenoid
H1	Unknown	Unknown	1.54 ± 0.02	1.38 ± 0.04	7.34 ± 0.05
H7	30º 56'	71º 31'	7.71 ± 0.22	5.62 ± 0.21	23.57 ± 0.53
H8	34º 04'	70º 56'	1.32 ± 0.04	2.54 ± 0.12	6.88 ± 0.22
H10	Unknown	Unknown	3.05 ± 0.10	1.03 ± 0.03	13.79 ± 0.26
H13	Unknown	Unknown	1.48 ± 0.05	1.27 ± 0.06	7.32 ± 0.16
H14	Unknown	Unknown	4.90 ± 0.13	1.87 ± 0.06	20.69 ± 0.44
H16	32º 18'	71º 31'	2.04 ± 0.08	0.63 ± 0.03	8.89 ± 0.24
H17	30º 54'	72º 22'	8.15 ± 0.17	5.23 ± 0.2	26.09 ± 0.35
H31	Unknown	Unknown	2.36 ± 0.03	1.32 ± 0.04	9.99 ± 0.05
H33	Unknown	Unknown	4.07 ± 0.07	2.67 ± 0.04	16.68 ± 0.28
H34	Unknown	Unknown	1.42 ± 0.03	0.68 ± 0.03	6.68 ± 0.08
H35	34º 04'	70º 56'	1.49 ± 0.01	2.36 ± 0.05	6.49 ± 0.06
H38	Unknown	Unknown	4.56 ± 0.02	2.17 ± 0.04	17.48 ± 0.10
H39	34º 03'	71º 38'	6.17 ± 0.15	2.18 ± 0.08	25.81 ± 0.43
H41	Unknown	Unknown	5.79 ± 0.07	4.93 ± 0.10	20.05 ± 0.21
H46	Unknown	Unknown	5.91 ± 0.13	3.88 ± 0.14	20.00 ± 0.32
H47	36º 45'	72º 18'	4.00 ± 0.08	2.53 ± 0.07	16.29 ± 0.32
H49	Unknown	Unknown	5.40 ± 0.09	3.39 ± 0.10	18.57 ± 0.31
H51	36º 45'	70º 34'	3.54 ± 0.12	2.23 ± 0.13	15.00 ± 0.35
H52	36º 45'	70º 34'	3.77 ± 0.02	2.46 ± 0.10	14.35 ± 0.10
H54	34º 51'	70º 34'	2.77 ± 0.05	2.09 ± 0.03	10.79 ± 0.13
H55	35º 51'	70º 34'	0.79 ± 0.02	*	15.71 ± 0.11
H56	Unknown	Unknown	5.46 ± 0.09	5.39 ± 0.08	18.48 ± 0.23
H57	34º 45'	70º 34'	1.26 ± 0.03	0.44 ± 0.02	7.97 ± 0.07
H58	35º 45'	71º 34'	1.93 ± 0.03	0.33 ± 0.01	13.09 ± 0.15
H59	36º 45'	72º 34'	1.08 ± 0.02	0.24 ± 0.01	7.67 ± 0.06
H60	37º 45'	73º 34'	1.09 ± 0.00	0.25 ± 0.01	6.84 ± 0.02
H61	Unknown	Unknown	3.78 ± 0.02	1.66 ± 0.05	14.54 ± 0.11
H68	36º 45'	72º 18'	8.23 ± 0.07	2.28 ± 0.03	35.72 ± 0.28
H74	33º 21'	71º 23'	5.24 ± 0.16	7.31 ± 0.24	19.14 ± 0.55
H75	Unknown	Unknown	1.74 ± 0.03	3.11 ± 0.03	8.91 ± 0.09
H83	Unknown	Unknown	1.68 ± 0.02	1.73 ± 0.01	6.44 ± 0.03
H93	33º 06'	71º 28'	2.26 ± 0.03	1.75 ± 0.01	8.36 ± 0.08
H200	34º 45'	70º 34'	2.17 ± 0.02	1.21 ± 0.02	8.92 ± 0.15
H202	33º 01'	70º 54'	2.67 ± 0.03	1.77 ± 0.03	10.52 ± 0.06
H203	32º 15'	71º 32'	2.65 ± 0.01	1.15 ± 0.00	10.59 ± 0.06
H204	33º 00'	70º 57'	1.63 ± 0.01	1.64 ± 0.00	6.86 ± 0.06
H205	32º 58'	71º 10'	5.76 ± 0.01	5.43 ± 0.07	19.01 ± 0.07
H206	33º 06'	71º 28'	1.76 ± 0.00	0.85 ± 0.02	7.93 ± 0.06
H207	31º 54'	72º 22'	7.71 ± 0.09	3.49 ± 0.12	28.04 ± 0.11
H208	32º 58'	71º 10'	0.94 ± 0.00	0.10 ± 0.01	6.58 ± 0.02
H209	33º 06'	71º 28'	2.10 ± 0.08	1.24 ± 0.05	8.66 ± 0.25
H210	33º 39'	70º 21'	3.49 ± 0.02	2.31 ± 0.05	12.53 ± 0.07
H211	32º 58'	71º 10'	0.72 ± 0.01	*	6.57 ± 0.03
H212	32º 15'	71º 32'	6.67 ± 0.05	2.22 ± 0.04	26.37 ± 0.28
H213	32º 25'	70º 55'	1.31 ± 0.02	0.36 ± 0.01	7.04 ± 0.05
H216	32º 18'	71º 31'	2.84 ± 0.01	0.54 ± 0.00	13.16 ± 0.04
H217	34º 04'	70º 56'	2.85 ± 0.06	8.92 ± 0.09	15.32 ± 0.19
H218	33º 04'	70º 57'	2.19 ± 0.01	3.07 ± 0.07	9.76 ± 0.03
H220	36º 45'	72º 18'	5.79 ± 0.23	6.77 ± 0.09	18.71 ± 0.31
H221	Unknown	Unknown	2.29 ± 0.01	0.94 ± 0.01	11.82 ± 0.16
H222	36º 45'	73º 09'	4.36 ± 0.01	2.64 ± 0.02	15.15 ± 0.08
H223	Unknown	Unknown	3.09 ± 0.04	1.45 ± 0.08	11.75 ± 0.20

Requena-Ramírez et al. Additional file 3. Geographic localization and carotenoid composition in 93 accessions of *H. chilense (data from Ávila et al. 2019. https://link.springer.com/article/10.1007%2Fs10681-019-2369-6)*

GENOTYPE	Latitiud	Longitud	Lutein monoesters	Lutein diesters	Total Carotenoid
H225	32° 18'	71° 31'	1.86 ± 0.02	2.07 ± 0.03	8.05 ± 0.07
H226	34° 03'	71° 38'	2.35 ± 0.01	2.02 ± 0.03	9.37 ± 0.03
H228	34° 04'	70° 56'	1.68 ± 0.01	0.63 ± 0.01	8.12 ± 0.06
H229	33° 38'	70° 18'	1.78 ± 0.01	0.48 ± 0.01	8.38 ± 0.05
H232	32° 25'	70° 55'	1.08 ± 0.01	0.31 ± 0.00	5.81 ± 0.06
H241	33'	70° 57'	1.59 ± 0.03	1.74 ± 0.01	6.87 ± 0.09
H245	34° 58'	70° 27'	4.84 ± 0.03	1.10 ± 0.01	20.18 ± 0.07
H250	38° 42'	73° 02'	2.49 ± 0.01	0.81 ± 0.00	11.43 ± 0.06
H251	38° 26'	71° 22'	6.25 ± 0.01	3.05 ± 0.01	25.16 ± 0.02
H252	38° 41'	73° 24'	5.00 ± 0.02	2.11 ± 0.02	17.30 ± 0.05
H254	34° 57'	70° 23'	2.35 ± 0.03	0.25 ± 0.01	16.51 ± 0.11
H255	38° 42'	73° 02'	3.37 ± 0.03	0.92 ± 0.01	16.02 ± 0.14
H261	30° 23'	70° 58'	7.57 ± 0.06	7.19 ± 0.12	23.73 ± 0.16
H266	30° 32'	71° 42'	3.05 ± 0.09	0.08 ± 0.01	43.06 ± 0.16
H283	30° 41'	70° 52'	1.11 ± 0.02	0.02 ± 0.00	19.62 ± 0.11
H286	29° 55'	71° 14'	7.79 ± 0.04	5.78 ± 0.07	23.17 ± 0.07
H290	30° 53'	71° 29'	*	*	31.35 ± 0.11
H292	30° 45'	71° 32'	6.31 ± 0.06	2.97 ± 0.03	23.54 ± 0.22
H293	30° 32'	71° 32'	3.47 ± 0.02	*	37.65 ± 0.10
H294	30° 37'	71° 19'	5.41 ± 0.05	1.49 ± 0.03	19.59 ± 0.12
H295	30° 41'	71° 22'	6.27 ± 0.03	6.23 ± 0.08	20.51 ± 0.11
H296	30° 41'	70° 52'	4.59 ± 0.01	1.42 ± 0.02	18.65 ± 0.06
H297	30° 32'	71° 29'	4.79 ± 0.01	1.03 ± 0.01	19.81 ± 0.09
H298	30° 21'	71° 29'	3.82 ± 0.09	1.38 ± 0.09	15.04 ± 0.20
H299	30° 15'	70° 41'	3.42 ± 0.03	0.59 ± 0.02	17.16 ± 0.14
H300	28° 55'	70° 45'	4.56 ± 0.04	1.04 ± 0.02	18.60 ± 0.13
H301	30° 41'	71° 22'	6.19 ± 0.17	5.89 ± 0.19	18.31 ± 0.31
H302	30° 41'	70° 51'	5.49 ± 0.09	1.07 ± 0.00	25.78 ± 0.44
H303	31° 54'	70° 22'	7.50 ± 0.10	2.35 ± 0.06	27.65 ± 0.10
H304	31° 48'	71° 21'	3.91 ± 0.04	3.45 ± 0.03	13.14 ± 0.12
H305	30° 37'	71° 14'	8.03 ± 0.04	4.75 ± 0.03	25.47 ± 0.14
H307	29° 55'	71° 14'	5.64 ± 0.15	6.71 ± 0.22	19.52 ± 0.39
H308	31° 47'	70° 35'	1.78 ± 0.01	0.36 ± 0.00	9.42 ± 0.02
H309	30° 37'	71° 14'	7.93 ± 0.13	7.95 ± 0.16	25.36 ± 0.35
H310	31° 56'	71° 31'	3.53 ± 0.03	*	26.51 ± 0.31
H311	30° 48'	71° 40'	3.71 ± 0.04	0.27 ± 0.03	24.57 ± 0.33
H313	34° 20'	71° 15'	1.77 ± 0.02	1.00 ± 0.01	7.21 ± 0.07
H315	36° 53'	73° 10'	5.27 ± 0.02	0.66 ± 0.01	27.43 ± 0.11
H317	34° 51'	70° 34'	3.31 ± 0.04	3.10 ± 0.03	11.15 ± 0.07
H319	Unknown	Unknown	5.48 ± 0.03	1.90 ± 0.03	20.68 ± 0.20

* No quantifiable amounts, referred as zero-esters
