# Supplementary material for: Mediation of a GDSL Esterase/Lipase in Carotenoid Esterification in Tritordeum Suggests a Common Mechanism of Carotenoid Esterification in Triticeae Species
Source: Front Plant Sci. 2020 Dec 17;11:592515. doi: 10.3389/fpls.2020.592515 (PMC7971304; doi:10.3389/fpls.2020.592515)
Supplement: Supplementary file 3 [file Data_Sheet_3.pdf]

Additional file 4. Requena-Ramírez et al.

Figure S2. Protein alignment of XAT-7Hch predicted protein in H290 accession of H. chilense and GDSL esterase/lipase- like proteins of different species (organism column), as shown by the ncbi BLASTp MSA viewer (Multiple sequence alignment, vs 1.18.1). Identical residues are shown as dots. The Gly residue at position 75 (Cys in XAT-7Hch_H290) is conserved in all proteins.

Sequence ID	Start	Alignment (38–124)	End	Organism
XAT-7Hch (290) (+)	1	SLVDVGNNNYIFTVAKANFPPYGRDFKDHVATGRFCNCKLLIDFIAEKIGFNSSPPAYLSPQASGRDLLLGANFASATSGYNDHGTL	352	
KAE8776963.1 (+)	1	.............I.......................G..........V................WN....................	352	Hordeum vulgare
KAF7045707.1 (+)	1	.............I.......................G...V......V...G............QN..........A.........	351	Triticum aestivum
XP_020178203.1 (+)	1	.............I.......................G..........V...G..L.....E...QN..........A.........	351	Aegilops tauschii subsp. ...
CAB3466997.1 (+)	23	.S......D.LH.II...........AN.........G..AT.IT.DTL..TTY.A.........QN..I.......G...Y..TA.	356	Digitaria exilis
CAD6340261.1 (+)	14	.T......D.LH.II...........AN.........G..AT.IT.DTL..TTY.A.........QN..I.......G...Y..TA.	360	Miscanthus lutarioriparius
XP_002436473.1 (+)	15	.T......D.LH.IL...........AN.........G..AT.IT.DTL..TTY.A.........QN..I.......G...Y..TA.	359	Sorghum bicolor
XP_025812097.1 (+)	23	.S......D.LH.II...........AN.........G..AT.IT.DTL..TTY.A.........QN..I.......G...Y..TA.	356	Panicum hallii
KAF0910786.1 (+)	17	.S......D.LH.II............NQ........G..AT.IT..TL..E.YA......E...KN..I.......G...Y..TA.	345	Oryza meyeriana var. gr...
XP_015642056.1 (+)	17	.S......D.LK.II............NQ.P......G..AT.IT..TL..E.YA......D...KN..I.......G...Y..TA.	345	Oryza sativa Japonica G...
XP_006655787.1 (+)	17	.S......D.LH.II............NGA.......G..AT.IT..TL..E.YA......E...KN..I.......G...Y..TA.	345	Oryza brachyantha
XP_004964566.1 (+)	23	.S......D.LH.II...........AN.........G..AT.IT.DTL..TTY.A.........QN..I.......G...Y..TA.	351	Setaria italica
OEL34345.1 (+)	19	.S......D.LH.II...........QN.........G..AT.IT.DTL..TTY.A.........QN..I.......G...Y..TA.	345	Dichanthelium oligosant...
XP_003557194.1 (+)	19	.S......D.LH.LI..D........QGR........G..AT.IT.DTL..T.Y.......E...QN..I.......G...Y..TA.	346	Brachypodium distachyon
AQK97696.1 (+)	27	.S......D.LH.II...........AN.........G..AT.IT.DTL..TTY.A.........QN..I.......G...Y..TA.	356	Zea mays
XP_008793255.1 (+)	33	.T......D..H.AF...C.......VN.Q.......G..AT.IT.NTL..T.Y...........KN..M.V.....A...Y.ETAY	352	Phoenix dactylifera
XP_020585086.1 (+)	36	.T......D..H.IF..D.........N.E.......G..AT.IT..TL..TTY.A.........KN..I.......A...Y.TAI.	355	Phalaenopsis equestris
PKA59004.1 (+)	45	.T........LP..F..D.........N.QP......G..AT.I...TL..TNF...........KN..I.......A...Y.NAI.	366	Apostasia shenzhenica
XP_020690263.1 (+)	13	.T......D.LH.IF..D.........N.E.......G..AT.IT..TL..TTF.V.........KN..I.......A...Y.TAI.	362	Dendrobium catenatum
XP_009388555.1 (+)	33	.T......D.LK..FR.D.........N.EP......G..AT.LT.DTL..TTY...........KN..I.......G...DETSY.	352	Musa acuminata subsp....
RWR77627.1 (+)	32	.A........LP..I..D........VT.KP......G..AT.IT..TL..TTF...........KN..I.V.....G...Y.KTA.	348	Cinnamomum micranth...
XP_010267093.1 (+)	1	.A......D.LA.IF..DY.......VN.E.......G..AT.IT..TL..STY.......K...KN..I.......G...D.KTS.	346	Nelumbo nucifera
XP_020251263.1 (+)	20	.S......D..T.FF..D.........NKQP......G..AT.IT..TL..T.Y...........KN..I.......G...Y..TAI	344	Asparagus officinalis
XP_002527431.1 (+)	32	.V..A....HLY.IV...........AN.KS......G..AS..T..N...T.Y......KE.E.TN..I......GA...DSTAK.	351	Ricinus communis
XP_012076167.1 (+)	18	.VI.A....HLY.IV...........IN.KP......G..AS..T..N...T.Y......KE.H.TN..I.......A...DTTAK.	343	Jatropha curcas
KAF6134324.1 (+)	27	.V.......GLY..V...........VS.SP......G..AT..T..NL..STY.K...N.E.K.KN..I.......S...S.TAN.	354	Kingdonia uniflora
XP_002271400.1 (+)	18	.A..A....HLD.IV...........IS.KP......G..AS..T..N...T.Y......KE.K.NN..I.......A...HTTAK.	345	Vitis vinifera
XP_031487707.1 (+)	5	.A.......HLY.LVR..........VT.SP......G..VT..T..SL..A.Y........G..KNI.I.......S...ERTAA.	331	Nymphaea colorata
KAF3341550.1 (+)	11	.S......D.LH.IF............NR........G..AT.IT..SL..TTY...........KN..I.......A...F.GTA.	326	Carex littledalei
PON81083.1 (+)	22	.V........RT.LF....Y......IN.Q.......G..AT.LT..TL..KTY.......E...KN..I.......A...DEKAAI	351	Trema orientale
XP_018811100.1 (+)	34	.A........LP.IF....Y......VN.QP......G..AT.IT..TL..KTY...........KN..I.T.....A...DENAAM	351	Juglans regia
KAF5192451.1 (+)	24	.V.......NLY.IV...........V..KPS.....G..AT.TG..N...T.Y......L..R.KR..MTT....GS...FYRAQ.	343	Thalictrum thalictroides
GFZ08086.1 (+)	19	.A......D.LP.IF....Y......TN.KP......G..AT.IT..TL..TTY...........KN..I.......A...FDKPAI	344	Actinidia rufa
KAF5185264.1 (+)	18	.V.......Q.Y.LVR..........VT.SP......G..AT..T..NL..TTY......SE.R.KN..I......GA...ENTAS.	343	Thalictrum thalictroides
PIN17096.1 (+)	26	.V......D..H.IF....Y.......NQE.......G..AT.IT..TL..TTY.........T.KN.........GA...D..TS.	343	Handroanthus impetigin...
OVA17898.1 (+)	26	.V.......QLY.LV...........VS.SP......G..AT..T..NL..T.Y.......E.K.KN..T.S.....A...Y.RAQ.	342	Macleaya cordata
XP_010925408.1 (+)	40	.T......D..H.IF....Y......VN.Q.......G..AT.IT.NTL..T.Y...........NN..M.V.....A...Y.GTAY	359	Elaeis guineensis
XP_023884990.1 (+)	7	.V.......NLY.LV...........VN.SP......G..AT..T..FL..T.Y......QE.T.KN..T.......S...Y.SAQ.	344	Quercus suber
OVA14459.1 (+)	38	.A..I...D.LP.IF..D........ANRKP......G..AT.IT..TL..KTY...........KN..I.......A...Y.KANS	357	Macleaya cordata
OWM73711.1 (+)	26	.V..A....GLL.IV...........VN.KP......G.TAA..T..N...T.Y......KK.RRNN..I.......A...Y.AAK.	347	Punica granatum
XP_015873524.2 (+)	35	.V.......NLY.IV....L......PN.KPS.....G..AS..T..NL..T.Y..P...SK.K.K...I.......A...Y.SAK.	355	Ziziphus jujuba
PIA43488.1 (+)	25	.V.......NLY.IVR..........V..KP......G..AT.TG..N...T.Y......L..R.KR..T.T....GS...FYRAQ.	344	Aquilegia coerulea
XP_010690130.1 (+)	26	.T......D.VA.LF..DY.......AN.KP......G..AT.IT.DTL..T.Y...........QN..I.......A...D.KTA.	354	Beta vulgaris subsp. vul...
KAF7814207.1 (+)	33	.A........LP.LF....Y......VN.QP......G..AT.IT..TL..K.YA..........KN..I.......A...DEKAAM	363	Senna tora
XP_020097905.1 (+)	13	.S......D.LH.IF....C.......N.........G..AT.IT..TL..T.YA..........KN..I.......A...D.TAN.	357	Ananas comosus
KZV39011.1 (+)	7	.V......D.VH.IF....Y.......N.E.......G..AT.IT..NL..T.Y.........T.KS..I......GA...D.RTS.	324	Dorcoceras hygrometric...
PON54498.1 (+)	22	.V........RT.LF....Y......IN.Q.......G..AT.LT..TL..KTY.S....SE...KN..I.......A...DEKAAI	351	Parasponia andersonii
PSR89900.1 (+)	23	.V..A....NLY.IV...........IQ.KS......G..AS..T..N...T.Y......KS...KN..I......GA...YEAAK.	342	Actinidia chinensis var. ...
KAF3774747.1 (+)	26	.A.......HLY.LVR..........VT.SP......G..VT..T..SL..A.Y........G..KN..I.......S...ERTAA.	331	Nymphaea thermarum
XP_007015991.1 (+)	28	.V..A....NLY.II...........VN.KP......G..AS..T..N...T.Y......K..E.KN..I.......S...YETAK.	347	Theobroma cacao
KAF3943444.1 (+)	55	.V.......NLY.LV...........VN.SP......G..AT..T..FL..T.Y......QE.T.KN..MT......S...Y.SAQ.	375	Castanea mollissima
XP_030926394.1 (+)	24	.V.......NLY.LV...........VNQSP......G..AT..T..FL..T.Y......QE.T.KN..T.......S...Y.SAQ.	344	Quercus lobata
PKU82394.1 (+)	13	.T......D.LH.IF..D.........N.E.......G..AT.IT..TL..TTF.V.........KN..I.......A...Y.-	347	Dendrobium catenatum
XP_015938449.1 (+)	30	.V......D.LP.IF....Y......VN.Q.......G..AT.IT..TL..K.YA......E...KN..I.......A...DENAAI	347	Arachis duranensis
GAV75932.1 (+)	24	.V........LV.LF....Y......PGRK.......G..AT.IT..FL..TTY...........KN..I.......A...D.NA..	344	Cephalotus follicularis
KAF8393468.1 (+)	36	.A........LL.LF..DY.......VN.QP......G..AT.IT..TL..TTY......S....KN..I.......A...Y.KTA.	351	Tetracentron sinense
XP_010504217.1 (+)	1	.V........LP.IFR.DY.......AN.KP......G..AT.IT..TL..TKY.......E...KN..I.......A...D.KAA.	347	Camelina sativa
XP_011072453.1 (+)	25	.V......D..H.IF....Y.......NQE.......G..AT.IT..NL..T.Y...........KN....S....GA...DE..TS.	342	Sesamum indicum
XP_021662537.1 (+)	24	.V..A....HLY.IV...........IN.QP......G..AS..T..N...T.Y......KE.Q.TN..I......GA...FYTAK.	343	Hevea brasiliensis
