# Supplementary material for: Mediation of a GDSL Esterase/Lipase in Carotenoid Esterification in Tritordeum Suggests a Common Mechanism of Carotenoid Esterification in Triticeae Species
Source: Front Plant Sci. 2020 Dec 17;11:592515. doi: 10.3389/fpls.2020.592515 (PMC7971304; doi:10.3389/fpls.2020.592515)
Supplement: Supplementary file 4 [file Data_Sheet_4.pdf]

Additional file 1. Requena-Ramirez et al.

**Table S1**. Set of homoeologues and orthologues genes from common wheat and *H. vulgare*, respectively, from the wheat 7D candidate genes for *Lute* locus.

| **Wheat 7D candidates** | **Wheat homoeologues** | **Barley orthologues** |
|---|---|---|
| TraesCS7D02G011600 | TraesCS4A02G481800 | HORVU7Hr1G001330 |
| | TraesCS7A02G014600 | |
| TraesCS7D02G037200 | TraesCS7A02G041500 | HORVU7Hr1G007590 |
| TraesCS7D02G093200 | TraesCS4A02G397000 | HORVU7Hr1G021310 |
| | TraesCS7A02G097100 | |
| TraesCS7D02G094000 | TraesCS4A02G397900 | HORVU7Hr1G021460 |
| TraesCS7D02G108900 | TraesCS7A02G113600 | HORVU7Hr1G024220 |

**Table S2.** Primers designed for amplification of the candidate genes in *H. chilense* and for RT-qPCR experiments.

| Gene | Amplification / sequencing | RT-qPCR |
|---|---|---|
| HORCH7Hg001330 | Fw-AGCGTTGGCTCTTCGTAAGG | Fw-CTGTGCCATTGTAAACCCTCC |
| | Rv-GCTCTTGAATGCCTTGAGCC | Rv-GACCCGACTTCGAACTGACCA |
| HORCH7Hg007590 | Fw-ACAACTCAAGATGGGCCCTG | Fw-GGCAACATGTTCCAGTTGAGGA |
| | Rv-AGTGATGCCAACCGTGCATA | Rv-AATAGGAGGTGGAGTGATAAGAAGG |
| HORCH7Hg21310 | Fw-CAGACCGTCGACCAGTTCTC | Fw-AGCGCAGCCGTCAGCTCT |
| | Rv-GTCGGCGATCACTTGGTTGG | Rv-CAGCATCCCTTCTTCGCCTC |
| HORCH7Hg021460 | Fw-AGTCTGGCAGGCTACAATGG | Fw-GAGCTACCGTTGACTCATTACG |
| | Rv-CAAAGTCACATGAACCGGCG | Rv-CCTCGGTGAAGCCTTGTGATA |
| | Fw-TACCAAGCTAAGCTGGCGG | |
| | Rv-TGAACCAATGTGGCATCAAGC | |
| | Fw-ATTCGGCACATCACGCTAGT | |
| | Rv-TCTGGAGTCCACAGGTCAGT | |
| HORCH7Hg024220 | Fw-GTCGGCGACAAGATCTGGAA | Fw-ATCCAGTGGTTCCAGAACCTG |
| | Rv-TGGGAGTAGAAGTCGGCGTA | Rv-CTCTGCCCGGCCACTTG |

**Table S3. BLASTn results using *H. chilense* sequences as queries versus *H. vulgare* and *T. aestivum* genomes at EnsemblPlants (http://plants.ensembl.org/).**

| Query | Genomic Location | Overlapping Gene(s) | Length | Score | E-val | %ID |
|---|---|---|---|---|---|---|
| HORCH7Hg001330 | chr7H:2944880-2945196 | HORVU7Hr1G001330 | 317 | 273 | 3.7E-151 | 96.5 |
| | chr7H:2944502-2944831 | HORVU7Hr1G001330 | 330 | 226 | 4.1E-123 | 92.1 |
| | chr7H:2945201-2945266 | HORVU7Hr1G001330 | 66 | 66 | 1.2E-27 | 100.0 |
| | 7D:5165878-5166263 | TraesCS7D02G011600 | 386 | 298 | 1.3E-165 | 94.3 |
| HORCH7Hg007590 | chr7H:9893237-9893270 | HORVU7Hr1G007590 | 34 | 22 | 0.22 | 91.2 |
| | chr7H:9893575-9893637 | HORVU7Hr1G007590 | 63 | 31 | 9.2E-7 | 87.3 |
| | chr7H:9892960-9893094 | HORVU7Hr1G007590 | 135 | 71 | 1.2E-30 | 88.1 |
| | chr7H:9893367-9893486 | HORVU7Hr1G007590 | 120 | 72 | 3.1E-31 | 90.0 |
| | chr7H:9893661-9893847 | HORVU7Hr1G007590 | 187 | 131 | 1.9E-66 | 92.5 |
| | 7D:18882747-18882782 | TraesCS7D02G037200 | 36 | 24 | 0.042 | 91.7 |
| | 7D:18882521-18882562 | TraesCS7D02G037200 | 42 | 34 | 4.5E-8 | 95.2 |
| | 7D:18882979-18883041 | TraesCS7D02G037200 | 63 | 35 | 1.1E-8 | 88.9 |
| | 7D:18882780-18882867 | TraesCS7D02G037200 | 88 | 48 | 2.0E-16 | 88.6 |
| | 7D:18882661-18882725 | TraesCS7D02G037200 | 65 | 57 | 8.4E-22 | 96.9 |
| | 7D:18882419-18882519 | TraesCS7D02G037200 | 101 | 77 | 9.8E-34 | 94.1 |
| | 7D:18883065-18883249 | TraesCS7D02G037200 | 185 | 129 | 9.1E-65 | 92.4 |
| HORCH7Hg021310 | chr7H:30199351-30199620 | HORVU7Hr1G021310 | 270 | 226 | 3.1E-123 | 95.9 |
| | chr7H:30199076-30199235 | HORVU7Hr1G021310 | 160 | 152 | 4.5E-79 | 98.8 |
| | chr7H:30199691-30199745 | HORVU7Hr1G021310 | 55 | 35 | 3.0E-9 | 90.9 |
| | 7D:55429660-55429950 | TraesCS7D02G093200 | 291 | 239 | 1.6E-130 | 95.5 |
| HORCH7Hg021460 | chr7H:30702633-30702961 | HORVU7Hr1G021460 | 329 | 249 | 1.0E-136 | 93.9 |
| | chr7H:30701740-30701997 | HORVU7Hr1G021460 | 258 | 230 | 2.2E-125 | 97.3 |
| | chr7H:30702093-30702326 | HORVU7Hr1G021460 | 234 | 210 | 1.9E-113 | 97.4 |
| | chr7H:30701413-30701604 | HORVU7Hr1G021460 | 192 | 132 | 6.7E-67 | 92.2 |
| | 7D:56402774-56402900 | TraesCS7D02G094000 | 127 | 55 | 1.8E-20 | 85.8 |
| | 7D:56402368-56402477 | TraesCS7D02G094000 | 110 | 62 | 1.2E-24 | 89.1 |
| | 7D:56402531-56402683 | TraesCS7D02G094000 | 153 | 121 | 7.3E-60 | 94.8 |
| | 7D:56403322-56403579 | TraesCS7D02G094000 | 258 | 138 | 5.3E-70 | 88.4 |
| | 7D:56402979-56403209 | TraesCS7D02G094000 | 231 | 155 | 3.8E-80 | 91.8 |
| | 7D:56403780-56403971 | TraesCS7D02G094000 | 192 | 160 | 3.9E-83 | 95.8 |
| HORCH7Hg024220 | chr7H:38486150-38486541 | HORVU7Hr1G024220 | 392 | 324 | 0.0 | 95.7 |
| | 7D:65600023-65600407 | TraesCS7D02G108900 | 385 | 321 | 1.8E-179 | 95.8 |
